# Estrogen Replacement Regulates Vaginal Innervations in Ovariectomized Adult Virgin Rats: A Histological Study

**DOI:** 10.1155/2017/7456853

**Published:** 2017-03-16

**Authors:** Ting Li, Yuanyuan Ma, Hong Zhang, Ping Yan, Lili Huo, Yongyan Hu, Xi Chen, Ting Li, Miao Zhang, Zhaohui Liu

**Affiliations:** ^1^Department of Obstetrics and Gynecology, Peking University First Hospital, 8 Xishiku Street, Beijing 100034, China; ^2^Animal Center Laboratory, Peking University First Hospital, 8 Xishiku Street, Beijing 100034, China; ^3^Department of Pathology, Peking University First Hospital, 8 Xishiku Street, Beijing 100034, China; ^4^Department of Nuclear Medicine, Peking University First Hospital, 8 Xishiku Street, Beijing 100034, China

## Abstract

*Background*. Our previous Gräfenberg spot findings confirmed that the distal-third areas of the anterior vaginal wall bore a significantly greater number of nerves and sexual hormone may have certain degree of influence on these significant differences. However, the role of estrogen in vaginal innervations remains controversial.* Methods*. To investigate whether hormonal-neural interactions occur in the vagina, sixty rats were randomly divided into six groups: Sham-operated, ovariectomy, and 4 treatment groups. After 2 weeks of treatment, vaginal biopsies were prepared with hematoxylin and eosin and PGP9.5 using immunohistochemistry.* Results*. The density of small nerve fibers was significantly higher in the distal-half areas of intact vaginal walls than the proximal-half areas (*P* = 0.001). In contrast, the overall PGP 9.5-ir fiber innervation density was significantly decreased in the OVX rats subjected to surgical menopause. Sustained estrogen administration for 2 weeks resulted in nerve fiber proliferation, with values reaching normal levels in the low-dose estradiol valerate group.* Conclusion*. Our findings indicate that systemic hormonal therapy with low-dose estradiol valerate is effective and safe for treating deficient vaginal innervation caused by low level of estrogen activity in menopausal women and may aid studies to identify an optimal estradiol dose to provide relief from vaginal discomfort.

## 1. Introduction

Low-estrogen levels have been implicated as the primary cause of female sexual dysfunction [1]. Menopause, which is characterized by declining ovarian function and reduced circulatory estrogen levels, is associated with a variety of adverse symptoms including vaginal dryness, irritation, itching, burning, and dyspareunia, indicating that reproductive hormones, especially estrogen, are vital for the normal maintenance of the female reproductive tract. These vaginal symptoms not only result from loss of hormonal trophic support for vaginal tissues, but also are a result of changes in vaginal innervation stemming from decreased estrogen availability [2].

Few studies have characterized female vaginal innervation such that tactile stimulation of the vagina can lead to orgasm [3]. Hilliges et al. first evaluated innervation of the human vaginal mucosa using protein gene product 9.5 staining in 1995 and reported that increased innervation in human distal and anterior vaginal wall compared to other sections [4]. We hypothesized that sexual responses, such as arousal, lubrication, orgasm, and pain, may be mediated by nerve fibers in the vaginal wall, and consequently the total nerve number would be associated with sexual dysfunction. A previous G-spot's study from our group examining human erotogenic nerves in the anterior vaginal wall revealed significant differences in the nerve fiber and microvessel densities when comparing distal-third and proximal-third vaginal areas in postmenopausal but not premenopausal women, suggesting that hormonal-neural interactions occur in the vagina [5].

However, the neurophysiology of the female vagina is poorly understood. Only a handful of studies have examined the relationship between estrogen and vaginal innervation. A study of human vaginal innervation by Griebling et al. showed that hormone replacement therapy (HRT) resulted in reduced overall vaginal innervation, with topical HRT being more effective than systemic HRT [6].

Additionally, studies have been using animal models to mimic human innervation changes. Alterations in the estrogen levels have been shown to affect vaginal innervation not only in humans, but also in experimental models. A rat study by Ting et al. found that pan-neuronal marker protein gene product 9.5-immunoreactive (PGP 9.5-ir) nerve fibers were increased by almost 50% (*P* < 0.001) 14 days after ovariectomy (OVX) and that this increase was completely reversed by one week of sustained exogenous estrogen administration [7]. Similar depletions in vaginal innervation were observed in rats following a sustained estrogen elevation at term pregnancy [8, 9]. In contrast, Pessina et al. reported that estrogen treatment and OVX did not significantly affect vaginal innervation, while testosterone was found to increase the numbers of small adrenergic nerve fibers [10], while Pelletier et al. showed a significant decrease in vaginal PGP 9.5-ir fibers following OVX [11]. Overall, the literature strongly indicates that the hormonal status can modulate vaginal innervation, but the role of estrogen in this process still remains unclear.

Our previous findings reported that estrogen treatment is effective for treating vulvovaginal atrophy caused by hypoestrogenism or advancing age in menopausal women [12]. In this present study, we hence investigated hormonal-neural interactions following estrogen treatment in the rodent vagina by quantifying neural density under conditions of supraphysiological dose (3.2 mg/kg/d and 1.6 mg/kg/d), physiological dose (0.8 mg/kg/d), subphysiological dose estradiol valerate (0.4 mg/kg/d), and ultralow estrogen induced by ovariectomy. In addition, we examine the effects of HRT on body weight as well as the entire length and wet weight of the vagina, which are not adequately addressed by previous studies in this area.

## 2. Methods

### 2.1. Animal Preparation

Sixty mature female Sprague-Dawley rats (2~3 months, weight: 225 ± 15 g) purchased from Beijing Vital River Laboratory Animal Technology Co. Ltd. (Beijing, China) were raised in Animal Center Laboratory of Peking University First Hospital. The animals received food and water ad libitum and were housed 4~5 per cage in a climate- and humidity-controlled environment (25 ± 1°C; relative humidity: 50%) using a 12 h : 12 h light/dark artificial cycle (lights on at 10:00 AM). The NIH guidelines for laboratory animal care were followed, and all experimental procedures and protocols were approved by the Ethics Committee of Peking University First Hospital (Beijing, China). All animal experiments should comply with the National Institutes of Health Guide for the Care and Use of Laboratory Animals (NIH Publications number 8023, revised 1978).

Sixty rats were randomly divided into six groups as follows: the Sham-operated (SHAM), the ovariectomy (OVX), and OVX + Bujiale® estradiol valerate (1 mg/tablet; Guangdong Branch of Bayer Healthcare, Guangdong, China) groups: E1, E2, E3, and E4, which received daily lavage treatments of 0.4 mg/kg, 0.8 mg/kg, 1.6 mg/kg, and 3.2 mg/kg of Bujiale Estradiol valerate, respectively. Daily injections of the 0.8 mg/kg lavage dosage for 2 weeks in OVX rats result in restoration of normal physiological estrogen levels [13]. Experimentation was initiated within a week of arrival. All rats were anesthetized by i.p. injection of 1% pentobarbital sodium (40 mg/kg), and then SHAM rats underwent sham surgery, that is, bilateral laparotomy without ovariectomies, while rats in the other five groups were bilaterally ovariectomized using the ventral approach [13]. Serum estradiol levels were examined using an immunoradiometric assay on postoperative day 28 in order to ensure successful OVX; that is, we verified that plasma estrogen levels were below the physiological range (according to Rusa et al., the baseline E_2_ in the rat: 17 ± 2 to 21 ± 2 pg/mL during various stages of the menstrual cycle [14]). Then different groups received the following treatments by lavage: once-daily administration of 10 mL/kg saline (SHAM and OVX groups), 0.4 mg/kg estradiol valerate (E1), 0.8 mg/kg estradiol valerate (E_2_), 1.6 mg/kg estradiol valerate (E3), and 3.2 mg/kg estradiol valerate (E4) for 2 weeks. All the drug doses were adjusted based on the weight of rats at the time of administration.

After 2 weeks of treatment, rats were anesthetized as described above. Whole vagina samples were collected from all groups as described by Ting et al. [7]. The entire vagina was transected at its junction with the cervix and then separated from adjacent connective tissue, from cervix to vulva, and removed as a single block that included the external vulva. The rat body weights, vaginal tissue weights, and total vaginal length were measured.

### 2.2. Tissue Processing

Freshly harvested vaginal tissues from sixty rats were cut transversely into two halves of equal length, immersed immediately in 10% neutral buffered formalin, and embedded in paraffin using conventional histopathological methods. Five-micron cryosections were obtained perpendicular to the longitudinal axis of the vagina from the center of two halves, where the smooth muscle and nerves are most fully developed [15].

### 2.3. Immunostaining for PGP 9.5

Each section was stained with hematoxylin and eosin (H&E) for routine microscopy. Slides were incubated with 3% peroxide-methanol at room temperature for 20 min to block endogenous peroxidase and with 1% diluted goat serum to block nonspecific binding sites. Next, we utilized immunohistochemical stains to improve visualization of vaginal nerves. Primary rabbit polyclonal antisera against PGP9.5 (ThermoFisher, Waltham, MA), a general cytoplasmic marker of neurons and certain neuroendocrine cells, and slides were incubated overnight at 4°C. Secondary staining was carried out using the avidin-biotin-peroxidase procedure, and slides were then counterstained with Gill's hematoxylin. Negative control sections were treated as described above, but the primary antibody was replaced by 0.01 M phosphate-buffered saline.

### 2.4. Quantitative Analyses of Vaginal Innervation

Five randomly selected sections from each immunostained set from each half of vaginal tissue were blind-coded and evaluated independently by two experienced pathologists using a digital microscope (BH2; Olympus, Tokyo, Japan). The total number of PGP9.5-ir neuronal profiles in the five selected sections (per 1.2 mm^2^) of each transverse section was quantified under high power (×40). Both longitudinal cross sections and radially oriented PGP9.5-ir nerves were counted. Taking into account differences in vaginal length, all the exact numbers of nerves were also adjusted by the length of the tissue block.

### 2.5. Statistical Analysis

Statistical analyses were carried out using SPSS 13.0 (SPSS, Chicago, IL, USA). Values are presented as the means ± standard deviation (SD). Data were compared by Student's *t*-test or one-way analysis of variance (one-way ANOVA). *P* < 0.05 was considered the cutoff for statistical significance.

## 3. Results

### 3.1. OVX May Lead to Reduced Vaginal Innervation

**Figure 1:**
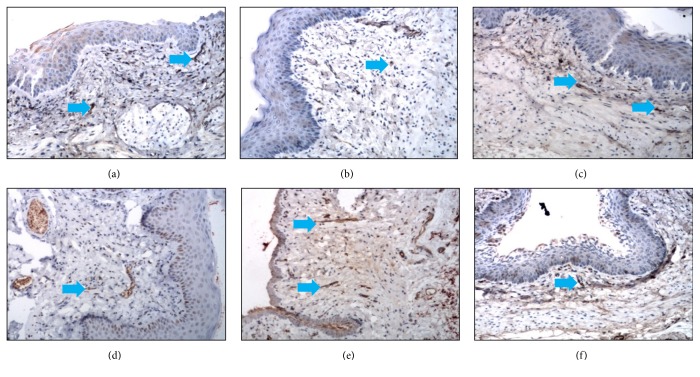
Immunohistochemical photomicrographs of sections taken from vaginal tissues. Sections were obtained from rats in SHAM group (a), OVX group (b), E1 (0.4 mg/kg/d (c)), E2 (0.8 mg/kg/d (d)), E3 (1.6 mg/kg/d (e)), and E4 (3.2 mg/kg/d (f)). Pan-neuronal marker PGP9.5 immunostaining: small nerve fibers are indicated by small blue arrows. The scale bar in (a)–(f) represents 50 *μ*m (200x magnification).

**Figure 2:**
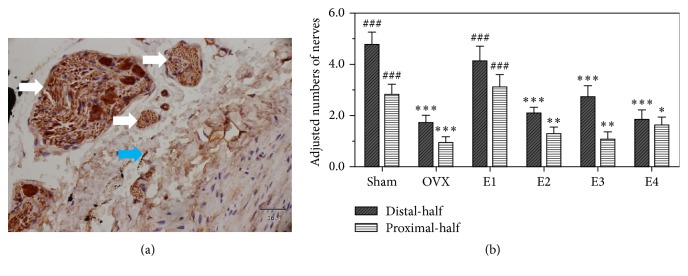
The effects of estrogen on vaginal innervation in rats. (a) Pan-neuronal marker PGP9.5 immunostaining: vaginal tissue sections from lamina propria layer of SHAM group rats. Nerve bundles are indicated by white arrows, while small nerve fibers are indicated by small blue arrows. Scale bar represents 30 *μ*m (400x magnification). (b) Quantitative analysis of nerve density in the rats' vagina: the Sham-operated (SHAM), the ovariectomy (OVX), E1 (0.4 mg/kg/d), E2 (0.8 mg/kg/d), E3 (1.6 mg/kg/d), and E4 (3.2 mg/kg/d) groups. *N* = 10 for each group. *∗* for *P* < 0.05; *∗∗* for *P* < 0.01; *∗∗∗* for *P* ≤ 0.001 versus SHAM group (control group) and ### for *P* < 0.001 versus OVX group (model).

The quantity and distribution of nerves in rats with SHAM treatment and OVX were examined (Figure 1, Table 1). While PGP 9.5 immunoreactive nerve fibers were observed uniformly in all the sections from the SHAM group rats, small nerve fibers were observed around arterial blood vessel walls and within the lamina propria and muscle layers of distal- and proximal-third areas (Figure 2(a)). Nerve bundles rarely appeared in the lamina propria and the muscle layer. Such PGP 9.5-ir fibers were rarely observed in the vaginal sections from intact control rats, unlike that previously observed in humans.

Compared to that in the SHAM-treated group, the overall innervation density of PGP 9.5-ir fibers in the distal- (*P* < 0.001) and proximal-half (*P* = 0.001) areas was significantly reduced in the OVX group (Figure 1, Table 1). The PGP 9.5-ir fibers observed in the SHAM group (*n* = 10) were small nerve fibers found in the lamina propria and muscularis of the distal- and proximal-half areas, while such fibers were rare within the vaginal epithelium (Figure 1(a)). Nerve bundles rarely appeared in the lamina propria, and those in the muscularis were unsuitable for a quantitative assessment. Quantitative analysis of SHAM group (intact control group) rats showed that density of small nerve fibers in the lamina propria and muscularis was significantly higher in the distal-half areas than the proximal-half areas (4.74 ± 0.51 versus 2.81 ± 0.41; *t* = 5.085, *P* = 0.001; paired samples *t*-tests).

### 3.2. Effect of Estrogen on Vaginal Innervation

After 2 weeks of sustained estradiol valerate administration, PGP 9.5-ir innervation in both the distal- and proximal-half areas was increased (by varying degrees) over that in the OVX rats (*P* < 0.001 for both areas; Figures 1 and 2(b), Table 2). The PGP 9.5-ir innervation density in both the distal- and proximal-half areas was significantly higher in the E1 group rats than in any other group (Table 2), returning essentially to control levels (*P* = 0.316  and  *P* = 0.525; Figures 1(c) and 2(b)). Importantly, the estradiol dose administered to the E1 group (0.4 mg/kg·d^−1^) is a subphysiological dose, and these results indicate that following such a low-dose administration, the increase in innervation induced by OVX was reversed, while this trend was not observed in rats treated with higher amounts of estradiol valerate.

## 4. Discussion

This study examining female SD rats demonstrates 3 important findings.

First, the rat may be a suitable model animal for studying human vaginal innervation. The overall density of intravaginal innervation is higher in distal-half areas of the vagina in female rats than the proximal-half areas, with immunoreactive nerve fibers being observed in the lamina propria and muscularis, but not the epithelial layer. These findings are in agreement with previous studies examining human vaginal innervation, whose networks of smaller nerve fibers were just below the epithelium and were more prevalent in the distal regions [4, 5].

Second, exogenous estrogen modulates vaginal innervation, and withdrawal of estradiol via ovariectomy significantly reduces the relative densities of the PGP9.5-ir nerves in female rats, and the observed neuroprotection is dose-dependent, requiring subphysiological estradiol level and long-term exposure to the hormone. While estrogen is known to regulate complex signaling pathways involved in neuroprotection in the central nervous system, estrogen-mediated neural regulation in female reproductive system, especially the vagina, is poorly defined. In ovariectomized mice, estradiol treatment resulted in a greater than 5-fold increase in the uterine expression of mature brain-derived neurotrophic factor (BDNF), pro-BDNF, and nerve growth factor receptor (NGFR) [16]. A review of estrogen-induced remodeling of reproductive tract innervation by Mónica Brauer and Smith [2] showed that estrogen depletion results in the rapid degeneration of sympathetic terminal axons in the myometrium, as well as vaginal autonomic and nociceptive axons, but such axons could regenerate under low-estrogen conditions. Impaired hormonal-neural control may result in a malfunction of the genital response including loss of desire, loss of genital sensitivity, loss of vaginal lubrication, erectile dysfunction, ejaculation disorder, and orgasmic disorder [17]. Additionally, they reported that unpublished results from this group showed a single acute injection of estrogen could deplete uterine innervation without altering vaginal innervation in adult OVX rats. This overview [2] illustrates that hormonally mediated neural plasticity is an extraordinarily complex phenomenon involving multiple target-derived and time-dependent mechanisms acting in concert to achieve selective reductions in innervation.

Interestingly, quantitative data from our study show that the innervation density in both the distal- and proximal-half vaginal areas decreased significantly after OVX as a result of the prolonged hypoestrogenic state in the vagina and diminished estrogen-mediated trophic support, and systemic estradiol administration resulted in regeneration. This is in agreement with data from Pelletier et al. [11] and Ahmed et al. [18] but differs from the results of Ting et al. [7] and Griebling et al. [6]. However, Pelletier et al. [11] just showed a significant decrease (59%) in vaginal PGP 9.5-ir fibers 9 months after OVX without further estrogen replacement therapy. Ahmed et al. [18] focused on estrogen recovering function on pudendal nerve crush, omitting vaginal innervation.

Numerous studies have delineated the neuroprotective effects of female sex hormones: the neuroprotective effects of estrogen have been well documented for Alzheimer's disease [19, 20], Parkinson's disease [21], spinal cord and brain injury [22–24], and so on, with the potential neuroprotective mechanisms including autoregulatory functions, antioxidant effects, reduction in excitotoxicity with glutamate exposure, increase in expression of the antiapoptotic factor bcl-2, and activation of mitogen-activated protein kinase pathways [25].

The distal-third area of the human vagina is mainly innervated by the pudendal nerve, resulting in a sensory nerve ending-dense area that is sensitive to sexual stimulus. Estrogen is capable of recovering and facilitating improvement in nerve regeneration following bilateral pudendal nerve crush in ovariectomized female rats [18]. This hormone thus exerts both direct and indirect trophic effects on total vaginal nerve distribution that outdistance degenerative effects on some types of nerve axons.

Ting et al. [7] studied innervation one week after OVX (with continuous estrogen treatment for that week), while Pelletier et al. [11] examined innervation in rats 9 months after OVX. In this study, examination of plasma estrogen levels one month after OVX revealed that they were below the lower limit of the physiological range. Taken in combination with the results from Ting et al. [7] and Pelletier et al. [11], these data indicate that the dynamic changes in vaginal innervation after OVX may involve a short-term compensatory increase followed by a sustained decline. Furthermore, the discrepancy may partly be the narrow therapeutic range of estradiol, because the benefit of estrogen in vaginal innervation is diminished at larger doses.

Menopause may damage nerve fibers within the lamina propria and muscularis and, in humans, may damage the possible G-spot leading to difficulty in sexual arousal or the achievement of orgasm and thus contribute to dyspareunia and associated issues. Estrogen may exert time-delayed neuroprotective effects, and it is likely that it may be neuroprotective only in a narrow therapeutic concentration range [14]. In this study, we observed complete restoration of vaginal innervation (to the control levels) only after treating with a subphysiological dosage (0.4 mg/kg/day) of estrogen. This finding underlines the importance of determining an appropriate neuroprotective dosage for successful hormone replacement therapy for menopausal women. Overall, for optimal neuroprotection, systemic hormone therapies with low-dose estradiol valerate or alternately topical hormone therapy would be more effective and safe for treating deficient vaginal innervation caused by a low level of estrogen activity in menopausal women.

This study, which examines innervation in the proximal and distal areas of the rodent vaginal wall, shows that the rat is a suitable model animal for studying human vaginal innervation. Systemic hormone therapy with low-dose estradiol valerate exerted neuroprotective effects on the vaginal nerve supply and may represent a more effective and safe option for treating urogenital atrophy and vaginal dysfunction without inducing endometrial proliferation in postmenopausal women [26]. Vaginal ER*α* expression was downregulated by estradiol, suggesting this may be a mechanism by which estrogen can influence vaginal function through direct or indirect effects on the vaginal target cells to prevent continued estrogenic stimulation. The present study will help gynecologists choose an appropriate hormone therapy and therapeutic dose for vaginal innervation repair, especially in menopausal women with female sexual dysfunction.

## Figures and Tables

**Table 1:** Vaginal innervation by location and comparisons of SHAM and ovariectomized (OVX) rats (alpha = 0.05, two-tailed).

Location	Subject *N*	PGP9.5-ir nerves (per 1.2 mm^2^)		*t*	*P*
		SHAM	OVX		
Distal-half	10	4.74 ± 0.51	1.72 ± 0.28	5.217	<0.001^*∗*^
Proximal-half	10	2.81 ± 0.41	0.93 ± 0.24	3.992	0.001^*∗*^

^*∗*^*P* < 0.05 (independent samples *t*-tests).

**Table 2:** Vaginal innervation by location and comparisons of all groups (per 1.2 mm^2^, alpha = 0.05, two-tailed).

Location	Subject *N*	SHAM	OVX	E1	E2	E3	E4	*F*	*P*
Distal-half	10	4.74 ± 0.51	1.72 ± 0.28	4.15 ± 0.55	2.06 ± 0.25	2.71 ± 0.44	1.83 ± 0.37	9.494	<0.001^*∗*^
Proximal-half	10	2.81 ± 0.41	0.93 ± 0.24	3.12 ± 0.47	1.28 ± 0.26	1.07 ± 0.29	1.61 ± 0.31	7.537	<0.001^*∗*^

^*∗*^*P* < 0.05 (one-way analysis of variance).
